# Antioxidant Activity of *Ixora parviflora* in a Cell/Cell-Free System and in UV-Exposed Human Fibroblasts

**DOI:** 10.3390/molecules16075735

**Published:** 2011-07-06

**Authors:** Kuo-Ching Wen, Hua-Hsien Chiu, Pei-Ching Fan, Chien-Wen Chen, Shih-Mei Wu, Jung-Hsiang Chang, Hsiu-Mei Chiang

**Affiliations:** 1 Department of Cosmecutics, China Medical University, 404, Taichung, Taiwan; 2 Center for Biomedical Technology Research and Development, Fooyin University, 831, Kaohsiung, Taiwan

**Keywords:** *Ixora parviflora*, antioxidant, metal chelating, reducing power, radical scavenging, reactive oxygen species

## Abstract

Polyphenols and flavonoids possess a variety of biological activities including antioxidant and anti-tumor activities. *Ixora parviflora* is a member of the flavonoid-rich Rubiaceae family of flowering plants and used as folk medicine in India. The aim of this study was to investigate the antioxidant activity of *Ixora parviflora* extract (IPE) in a cell-free system and erythrocytes, and the ability of IPE to inhibit reactive oxygen species (ROS) generation in human fibroblasts (Hs68) after ultraviolet (UV) exposure. Various *in vitro* antioxidant assays were employed in this study. The extraction yield of IPE was 17.4 ± 3.9%, the total phenolic content of IPE was 26.2 μg gallic acid equivalent (GAE)/mg leaves dry weight and the total flavonoids content was 54.2 ± 4.4 μg quercetin equvalent (QE)/mg extract. The content of chlorogenic acid was 9.7 ± 1.2 mg/g extract. IPE at 1000 μg/mL exhibited a reducing capacity of 90.5 ± 0.6%, a 1,1-diphenyl-2-picrylhydrazy (DPPH) radical scavenging activity of 96.0 ± 0.4%, a ferrous chelating activity of 72.2 ± 3.5%, a hydroxyl radical scavenging activity of 96.8 ± 1.4%, and a hydrogen peroxide scavenging activity of 99.5 ± 3.3%. IPE at 500 μg/mL also possessed inhibitory activity against 2,2′-azobis (2-methylpropionamidine) dihydrochloride (AAPH)-induced hemolysis of erythrocytes (89.4 ± 1.8%) and resulted in a 52.9% reduction in ROS generation in UV-exposed fibroblasts. According to our findings, IPE is a potent antioxidant and a potential anti-photoaging agent.

## 1. Introduction

High levels of reactive oxygen species (ROS) such as superoxide anion (O_2_^−^), hydroxyl radical (OH^·^), and hydrogen peroxide (H_2_O_2_) can cause oxidative damage to cellular DNA, protein and lipids, resulting in the initiation or development of numerous diseases such as cancer, cardiovascular diseases, type 2 diabetes mellitus, cataracts, rheumatoid arthritis, and various neurodegenerative diseases [1,2]. In addition, free transition metal ions cause extensive oxidative damage to cellular biomolecules such as DNA and proteins [3]. Biochemical reactions and exposure to numerous environment stresses such as pollutants and ultraviolet (UV) irradiation can generate ROS. UV irradiation is a ubiquitous hazardous factor causing skin damage through two different pathways. One is by direct absorption of UV *via* cellular chromophores, which triggers a series of chemical reactions. The other pathway results in the generation of ROS, which react with macro-molecules like DNA, proteins, and lipids, resulting in oxidative stress and photoaging [4,5]. Antioxidant defenses exist endogenously in the form of superoxide dismutases, catalases, and glutathione peroxidases and can be obtained from foods or dietary supplements such as vitamin E, vitamin C, carotenoids, thiol compounds and phenolic acids, and its subgroup, flavonoids (including flavones, isoflavones, flavonones, anthocyanins, catechins, and isocatechins) [1]. 

A large number of plants and phytoconstituents are potent scavengers of excess ROS and have been shown to have protective effects against the development of tumours [1,6], inflammatory [7,8,9], neurological and cardiovascular diseases [1,9,10], as well as environmental damage caused by pollutants and UV irradiation [11,12]. *Ixora parviflora* belongs to the flavonoid-rich Rubiaceae flowering plant family. This plant is used as a folk medicine in India and is also found distributed in China and Taiwan. The leaves were reported to be used in diarrhea [13], to check haemorrhages in child birth, eczema and skin diseases [14,15]. Water and methanol extracts of *Ixora parviflora* showed high antifungal activity against *Candida albicans* and *Aspergillus niger* [16]. Polyphenols and flavonoids possess a variety of biological activities including antioxidative and inhibitory effects that can protect against or minimize photoaging of the skin. In recent years, natural antioxidant research has been notably increased for finding safe and efficient agents ameliorating the adverse effects of ROS. The aim of this study was to investigate the antioxidant activity of *Ixora parviflora* extract (IPE) in cell and cell-free systems, and in addition, the ability of IPE to inhibit ROS generation in human fibroblasts (Hs68) after UV exposure.

## 2. Results and Discussion

### 2.1. Results

#### 2.1.1. The Extraction Yield and Quantation of IPE

The extraction yield of *Ixora parviflora* leaves was 17.4 ± 3.9%. The total phenolic content in the extract was determined by the Folin-Ciocalteu method. The regression coefficient of the calibration curve was 0.9993. The total phenolic content was 26.2 μg GAE/mg. The total flavonoids content was determined according as the aluminum chloride colorimetric method. The regression coefficient of the calibration curve was 0.9920 and the total flavonoids content was 54.2 ± 4.4 μg QE/mg extract. The amount of chlorogenic acid in the extract was 9.7 ± 1.2 μg/mg by a HPLC-DAD method.

#### 2.1.2. The Physical Characteristics of IPE

**Figure 1:**
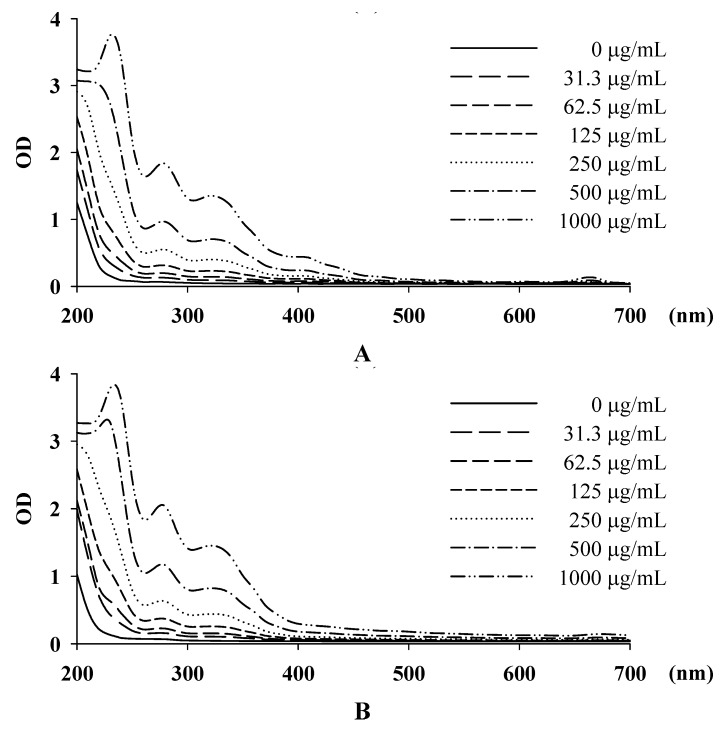

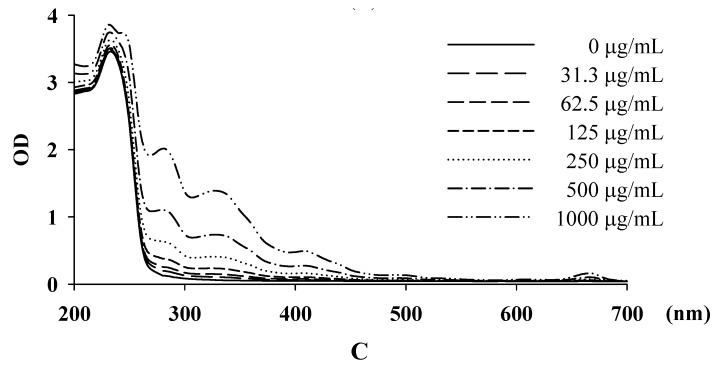
Absorption spectra of IPE in different solvent. (**A**) MeOH; (**B**) 50% PG and (**C**) DMSO.

The pH value of IPE was 4.94 ± 0.08 in distilled water, 5.04 ± 0.10 in methanol, 4.76 ± 0.08 in DMSO, and 6.67 ± 0.06 in 50% propylene glycol. The pH of human skin is 5.5 and the pH value of IPE was appropriate for skin application. The absorption spectrums of IPE in different solvents are shown in Figure 1. 

The absorption increased as the concentration increased and the maximum absorption was 210-250 nm for IPE in water, 260-290 nm for IPE in methanol, 300-350 nm for IPE in DMSO, and 400-410 nm for IPE in 50% propylene glycol. The absorption spectrum of IPE was in UVA and B range and IPE may be a UV absorber. 

#### 2.1.3. Measurement of Reducing Power

**Figure 2:**
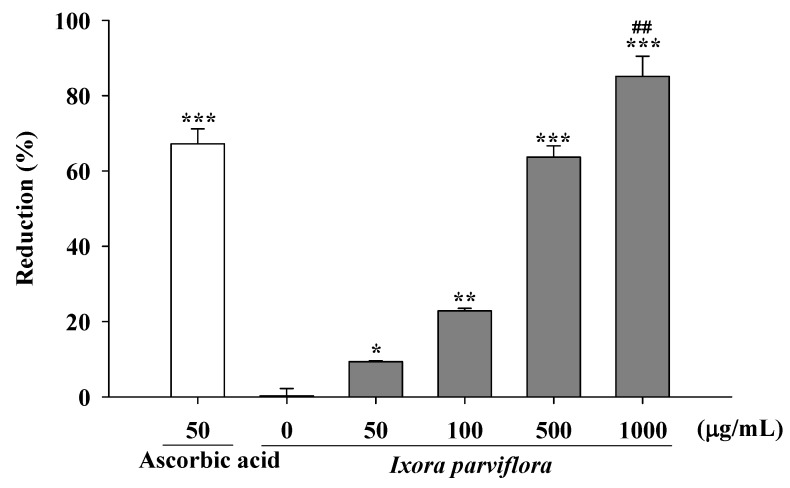
Reducing capacity of IPE. Ascorbic acid was applied as positive control. (n = 4; significant difference versus control (without extract): ^*^, *P* < 0.05; ^**^, *P* < 0.01; ^***^, *P* < 0.001; significant difference versus ascorbic acid: ^##^, *P* < 0.01).

The reducing capacity of IPE was 11.0 ± 0.5% at 50 μg/mL, 23.9 ± 0.6% at 100 μg/mL, 64.2 ± 3.0% at 500 μg/mL, and 90.5 ± 0.6% at 1000 μg/mL (Figure 2). The reducing capacity of the positive control (50 μg/mL ascorbic acid) was 79.1 ± 2.2%.

#### 2.1.4. DPPH Radical Scavenging Activity of IPE and IPH

**Figure 3:**
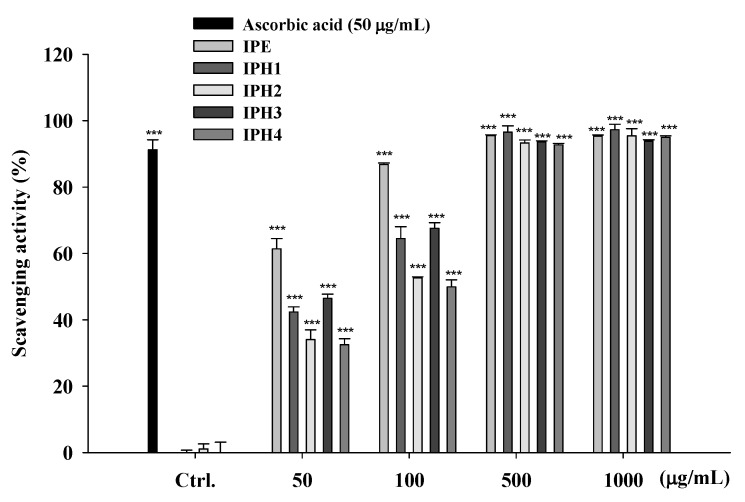
DPPH radical scavenging activity of IPE and its hydrolysates. (n = 4; ^***^, *P* < 0.001).

DPPH, which is stable and accepts an electron or hydrogen radical to become a stable diamagnetic molecule, is used as a reagent to evaluate free radical scavenging activities of antioxidants. Figure 3 shows the free radical scavenging activity of IPE (50-1000 μg/mL), IPH (50-1000 μg/mL) and ascorbic acid (50 μg/mL). The results indicated that IPE and IPH showed scavenged DPPH free radical in a dose-dependent manner. We found that IPE exhibited a scavenging activity of 66% at 50 μg/mL and the activity was 90% at doses higher than 100 μg/mL. The DPPH scavenging activities of the hydrolysates at 500 μg/mL were 94.3 ± 0.2% for IPH1, 89.5 ± 0.2% for IPH2, 92.7 ± 0.1% for IPH3, and 92.0 ± 0.4% for IPH4. All IP preparations exhibited similar DPPH free radical scavenging activities as ascorbic acid did.

#### 2.1.5. Metal Chelating Activity

**Figure 4:**
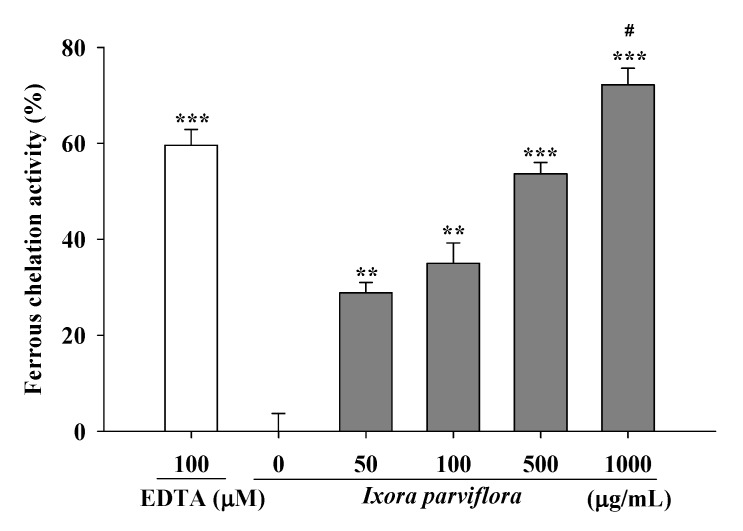
Ferrous chelation activity of IPE. EDTA was applied as positive control. (n = 4; significant difference versus control (without extract): ^**^, *P* < 0.01; ^***^, *P* < 0.001; significant difference versus EDTA: ^#^, *P* < 0.05).

Figure 4 shows the metal chelating activities of IPE and the positive control, EDTA. The activities of various concentrations of IPE (50, 100, 500 and 1,000 μg/mL) ranged from 28.9 ± 2.2% to 72.2 ± 3.5% and that of EDTA (100 μM) was 59.6 ± 3.3% (Figure 4). The activity of IPE on the metal chelating was significantly higher than EDTA at 1,000 μg/mL. These results showed that the ferrous ion chelating effect of IPE was statistically higher than EDTA at 1,000 μg/mL. It indicated IPE had effective ferrous ion chelating capacity.

#### 2.1.6. Determination of Hydroxyl Radical Scavenging Activity

**Figure 5:**
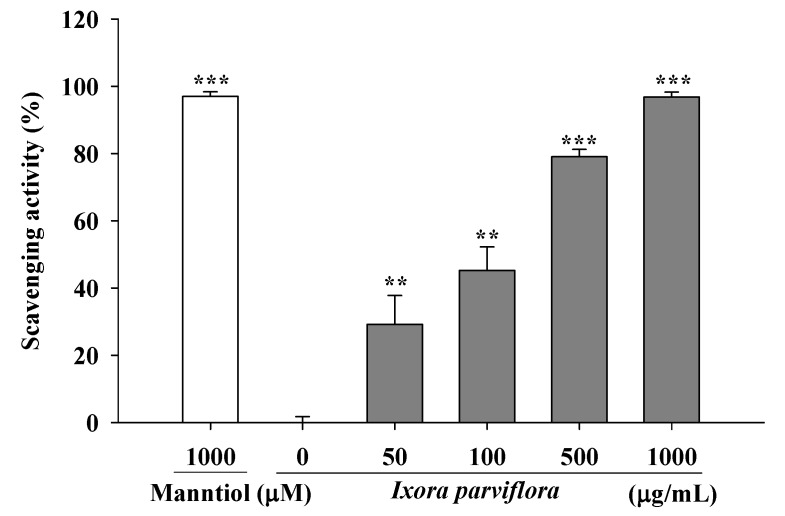
Scavenging hydroxyl radical by IPE. (n = 4; significant difference versus control (without extract): ^**^, *P* < 0.01; ^***^, *P* < 0.001).

The hydroxyl radical scavenging activities of IPE and the positive control mannitol are shown in Figure 5. The hydroxyl radical scavenging activity of various concentrations of IPE (50, 100, 500 and 1,000 μg/mL) ranged from 29.2 ± 8.6% to 96.8 ± 1.4% and that of mannitol (1,000 μM) was 97.0 ± 1.4%.

#### 2.1.7. Peroxide Scavenging Assay

**Figure 6:**
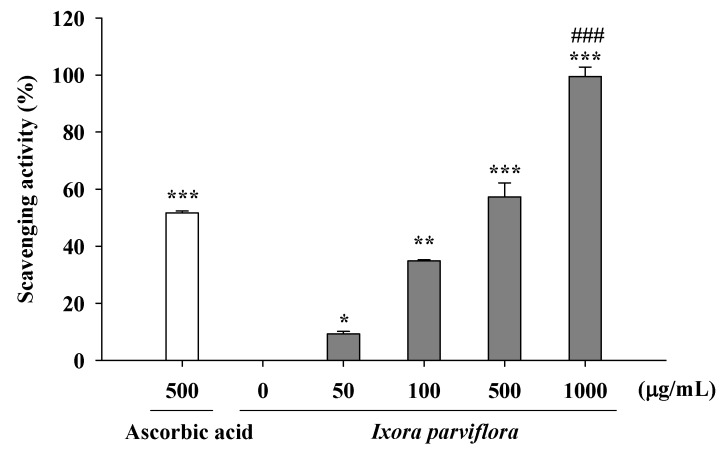
Scavenging hydrogen peroxide by IPE. (n = 4; significant difference versus control (without extract): ^*^, *P* < 0.05; ^**^, *P* < 0.01; ^***^, *P* < 0.001; significant difference versus ascorbic: ^###^, *P* < 0.001).

Peroxide is the primary product of oxidation produced during the initial stage of oxidation. The peroxide scavenging activities of IPE and the positive control ascorbic acid are shown in Figure 6. The hydroxyl radical scavenging activity of various concentrations of IPE (50, 100, 500 and 1,000 μg/mL) ranged from 9.2 ± 1.0% to 99.5 ± 3.3% and that of ascorbic acid (500 μg/mL) was 51.3 ± 0.1%. The activity of IPE was similar to equal concentration (500 μg/mL) of ascorbic acid.

#### 2.1.8. The Inhibitory Effects of IPE and IPH on AAPH-Induced Erythrocyte Hemolysis

**Figure 7:**
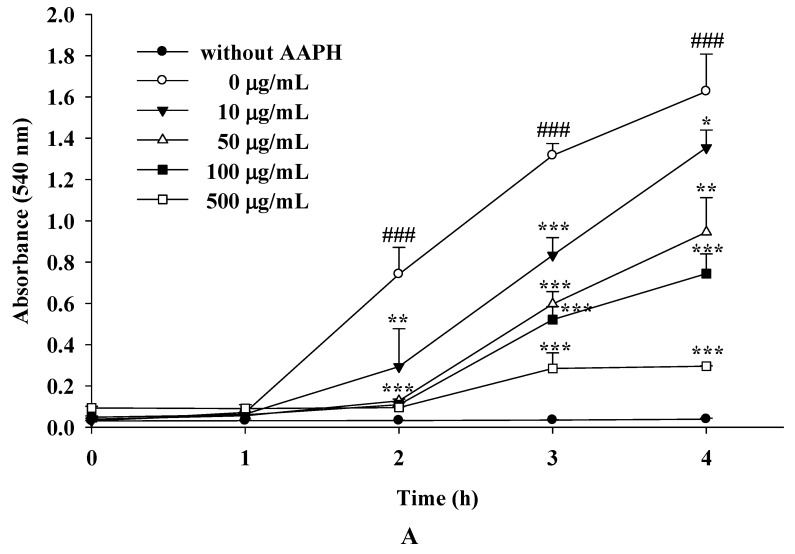

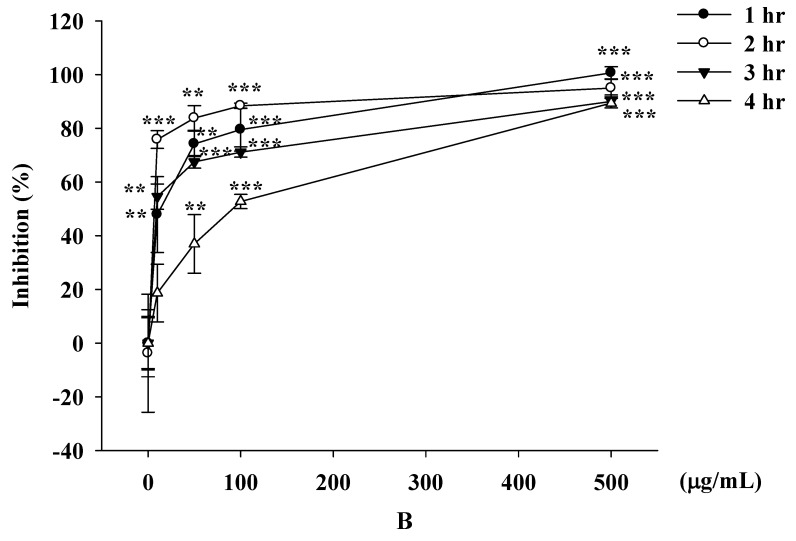
Inhibitory activity of IPE against AAPH-induced hemolysis of erythrocytes. (n = 4; significant difference versus control (without AAPH): ^###^, *P* < 0.001; significant difference versus control (without extract): ^*^, *P* < 0.05; ^**^, *P* < 0.01; ^***^, *P* < 0.001).

Biomembranes with a high content of polyunsaturated fatty acids, such as those of red blood cells, are easily damaged by free radicals [17]. AAPH-induced hemolysis is a common model for studying biomembrane damage [18,19]. In this study, we found that the erythrocytes did not exhibit hemolysis without AAPH. In addition, hemolysis occurred only after incubating erythrocytes with AAPH for more than 2 h, and the degree of AAPH-induced hemolysis was time dependent (Figure 7A).

Erythrocytes incubated with IPE at 10 μg/mL for 2 h exhibited a significant reduction in the rate of AAPH-induced hymolysis (Figure 7A. The protective effect of IPE on AAPH-induced hemolysis was dose dependent (Figure 7B). These results indicated IPE exhibited a potent AAPH free radical scavenging effect.

#### 2.1.9. Fluorescence Assay of Intracellular ROS

DCFDA staining and fluorescence microscopy were used to qualitatively characterize the degree of ROS generation. Fibroblasts were exposed to UVB (80 mJ/cm^2^) and then incubated with 10 μM of DCFDA for 30 min in a 24-well plate. After removing the DCFDA-containing medium, the cells were washed with PBS and treated with IPE for 24 h. 

**Figure 8:**
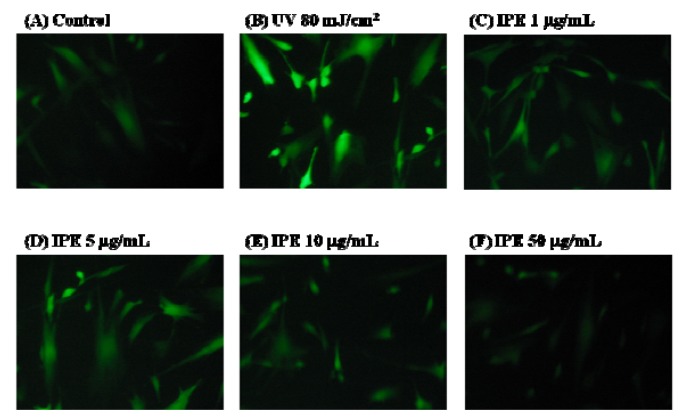

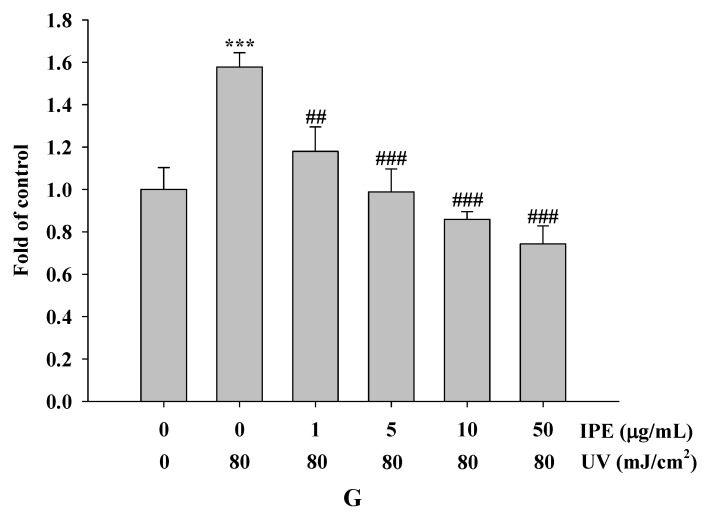
Repressive effect of IPE on intracellular oxidative stress in UV-irradiated Hs68 cell. (n = 4; significant difference versus control (without UV exposure and treatment): ^***^, *P* < 0.001; significant difference versus non-treatment group: ^##^, *P* < 0.01; ^###^, *P* < 0.001).

As shown in Figure 8B and Figure 8G, ROS levels were markedly higher in UVB-exposed fibroblasts than in control cells; however, this increase in ROS generation was attenuated in a dose-dependent manner in UVB-exposed fibroblasts that had been pretreated with various concentrations of IPE (1-50 μg/mL) (Figure 8C-G).

### 2.2. Discussion

The major findings of this study were that IPE showed: (1) high reducing capacity; (2) free radical scavenging; (3) significant metal chelating activity; and (4) ameliorating of UVB-induced ROS. According to our results, we expect IPE may be useful in treating aging and photo-aging related disease, possibly due to its high total phenolic and total flavonoids content. Plants biosynthesize and accumulate a variety of bioactive secondary metabolites such polyphenols and anthocyanidin. It has been reported that plants with high total phenolic content have been shown to exhibit excellent antioxidant effects [20]. In addition, studies have shown that the free radical scavenging effect is dependent on the total phenolic content. For example, *Cuminum cyminum* oil, which has a total phenolic content of 33.4 μg GAE/mg, exhibited a dose-dependent scavenging of DPPH radicals, and 5.4 μg of the oil was sufficient to scavenge 50% of DPPH radicals per mL [21]. *Trigonella foenum-graecum* (33.3 μg GAE/mg) and *Citrus sinensis* (29.0 μg GAE/mg) have been shown to scavenge more than 60% of DPPH radicals at a dose of 50 μg/mL compared with the 43% scavenging activity of *Spinacia oleracea*, a compound with a phenolic content of only 16.9 μg GAE/mg [22]. Bae *et al*. found that *Sorbus commixta*, with a total phenolic content of 447.3 μg GAE/mg, exhibited powerful free radical scavenging effects and decreased MMP-1 mRNA expression in human dermal fibroblasts [20]. The studies mentioned above showed the antioxidant activity was related to the total phenolic contents. Crude extracts of plant materials rich in phenolics are increasingly of interest in the food, health food and cosmetic industries because of the health benefits. The total phenolic and flavonoids content assay may be a fast and convenient method for screening phenol-rich materials. In this study, IPE, with a total phenolic content of 26.2 g GAE/mg and a total flavonoids content of 54.2 ± 4.4 μg QE/mg, was capable of scavenging more than 66% of DPPH free radicals and preventing AAPH-induced hemolysis at a dose of 50 μg/mL. In addition, the reducing power of IPE at 100 μg/mL was 23.9%, similar to quercetin [23], the antioxidant standard, indicating that IPE is a potent antioxidant. The HPLC-DAD result found the major component of IPE is chlorogenic acid. It was widely reported that chlorogenic acid and derivatives are well known to be antioxidants [24,25]. Therefore, chlorogenic acid and related compounds may be the major contributor to the antioxidant activity of IPE extract.

Iron is an important metal in biosystems; however, ferrous ion can react with H_2_O_2_ to produce the free radical OH^·^ leading to oxidative stress. The ability of a substance to chelate iron would be a valuable antioxidant property. In addition, iron with high reactivity is the most important factor affecting lipid peroxidation in transition metal. Ferrous chelation may render important antioxidant effects by retarding metal catalyzed oxidant. Results of the antioxidant assays in this study showed that IPE chelated 72.2% of free Fe^2+^, scavenged 99.5% of the available H_2_O_2_, and scavenged 96.8% of the hydroxyl radicals. The results indicate that IPE is a powerful antioxidant. Plant polyphenols are known to inhibit lipid peroxidation by quenching lipid peroxy radicals and reducing or chelating iron in the cell, thus preventing initiation of liplid peroxidation [26]. In our study, IPE exhibited potent free radical scavenging and ferrous ion chelating activities. Based on these results, we expect IPE could be applied to ameliorate the incidence of aging related disease. 

Polyphenols are predominantly present as glycosides in Chinese herbs, and it has been reported that aglycone structures are more important than glycone structures in conferring antioxidant activity because of the free hydroxyl group [27,28]. Not all studies, however, have found this to be true. For example, although the antioxidant activity of baicalein was shown to be superior to its glycoside, baicalin [29], the DPPH free radical scavenging activity of luteolin was not better than that of luteolin 7-O-glucoside [30]. Similarly, we found that the DPPH free radical scavenging activity of IPH was not superior to that of IPE. 

High concentrations of reactive oxygen species, including superoxide anion, hydrogen peroxide, and hydroxyl radical, can induce various human diseases. In this study, AAPH was used as the free radical and rat erythrocytes served as the biomembrane. We found that IPE protected against AAPH-induced injury in a dose-dependent and time-dependent manner. In addition, we used DCFDA as a probe to study the effect of IPE on UV-induced intracellular ROS generation in fibroblasts. DCFDA, a compound with two hydrophobic groups, passes through the cell membrane where it is hydrolyzed in cytosol by esterase into the hydrophilic compound DCF. That molecule then reacts with ROS to form a fluorescent substance. The intensity of the fluorescence is proportional to the concentration of ROS generated. The results indicated that IPE attenuated the UV-induced generation of ROS, and were consistent with the results of the reducing power, metal chelating, and DPPH scavenging assays. The antioxidant activities of IPE in the cell-free system were consistent with the results in erythrocyte and fibroblast models. The antioxidant activity of IPE is mainly due to its redox properties, which allow it to act as a reducing agent, hydrogen donor, and singlet oxygen quencher and metal chelating activity. UV exposure can lead to the depletion of endogenous antioxidants resulting in ROS-induced aging and DNA damage, especially in skin. IPE with UV-induced ROS quenching effect and UV absorber may be applied in cosmetic industries. As a consequence, the potential ROS quenching and photo-protective effects of dietary antioxidants from natural sources such as IPE warrant further studies. 

## 3. Materials and Methods

### 3.1. Materials

Hydrochloric acid, methanol, dimethyl sulfoxide (DMSO), chlorogenic acid, gallic acid, quercetin, aluminum chloride hexahydrate (AlCl_3_), potassium acetate (CH_3_COOK), calcium chloride (CaCl_2_), propylene glycol (PG), DL-dithiothreitol, Folin-Ciocalteu reagent, 1,1-diphenyl-2-picrylhydrazyl radical (DPPH) and 2,2′-azobis(2-methylpropionamidine) dihydrochloride (AAPH), FeCl_2_, 2-thio-barbituric acid (TBA), 2′,7′-dichlorofluorescin diacetate (DCFDA), 3-(2-pyridyl)-5,6-diphenyl-1,2,3-triazine-4′,4′′-disulfonic acid sodium salt (Ferrozine^®^), butanol, pyridine, sodium nitrite, trichloroacetic acid (TCA), and deoxyribose were purchased from Sigma-Aldrich Chemicals (St. Louis, MO, USA). Fetal bovine serum (FBS), penicillin-streptomycin, trypsin-EDTA, and Dulbecco’s Modified Eagle’s Medium (DMEM) were purchased from Gibco, Invitrogen (Carlsbad, CA, USA). Human foreskin fibroblasts were obtained from the Bioresource Collection and Research Center (Hsinchu, Taiwan).

### 3.2. Preparation of Ixora Parviflora Extract (IPE) and Its Hydrolysates (IPH)

The leaves of *Ixora parviflora* (IP) were harvested in Changhua County, Taiwan. Fresh leaves were dried in an oven at 50 °C. The dried leaves were ground and then extracted twice with a 30-fold volume of methanol ultrasonically for 1 h. The supernatant was filtered and the filtrate was collected. The filtrate was evaporated to dryness in a vacuum system. IPE was dissolved in DMSO and then hydrolyzed in the presence of 1.2 N HCl (2 mL) at 80 °C for 60 min and 2.4 N HCl (2 mL) at 100 °C for 60 min. After hydrolysis, the solution was partitioned with ethyl acetate (EA). The EA layer was evaporated to dryness in vacuum. The abbreviations and hydrolytic conditions of *Ixora parviflora* hydrolysates (IPH) are as follows: IPH1, 1.2 N HCl at 85 °C; IPH2, 2.4 N HCl at 85 °C; IPH3, 1.2 N HCl at 100 °C; IPH4, 2.4 N HCl at 100 °C. IPE and its hydrolysates were stored at −20 °C before use.

### 3.3. Quantation of IPE

#### 3.3.1. Total Phenolic Content of IPE

The total phenolic content of IPE was determined by the Folin-Ciocalteu reaction with slight modification as described previously [31]. Briefly, a mixture of IPE, Folin-Ciocalteu phenol reagent, and sodium carbonate was prepared and allowed to stand at room temperature for 30 min. After that, the mixture was centrifuged and the supernatant was measured at 760 nm. Gallic acid was used as the standard for the calibration curve. The phenolic contents were calibrated using a linear equation based on the calibration curve. The contents of phenolic compounds are expressed as μg gallic acid equivalent (GAE)/mg IP leaves dry weight.

#### 3.3.2. Total Flavonoids Content of IPE

The total flavonoids content was determined according as the aluminum chloride colorimetric method with slight modification [32]. Briefly, IPE was dissolved in methanol to afford a 100 μg/mL sample solution. The sample solution (200 μL) was mixed with 95% ethanol (600 μL), 10% aluminum chloride hexahydrate (AlCl_3_, 40 μL), 1 M potassium acetate (CH_3_COOK; 40 μL) and deionized water (520 μL). The mixture was at room temperature for 40 min, the reaction mixture absorbance was measured at 405 nm against a deionized water blank in an ELISA reader (Tecan, Austria). Quercetin was used as the standard for the calibration curve. The levels of total flavonoids contents in IPE were determined in triplicate and the result was expressed as microgram quercetin equivalents (QE)/mg extract. 

#### 3.3.3. Quantation of IPE by High Performance Liquid Chromatography-Diode Array Detector (HPLC-DAD) Method

The HPLC system consisted of a Hitachi EliteLaChrom, a Hitachi L-2130 pump, a Hitachi L-2200 autosampler and a Hitachi L-2450 diode array detector (DAD). Samples were separated by a reverse-phase column (Phenomenex C18, 4 μm, 4.6 × 250 mm) maintained at 25 °C. The flow rate was 1 mL/min. The detection wavelength was set at 280 nm. The elution was conducted in gradient fashion of solvent A (0.04% acetic acid) and solvent B (methanol), starting at 20% methanol for 6 min, increasing methanol to 60% at 11 min, reducing methanol to 30% at 26 min and reducing methanol back to 20% at 34 min as previous study described [33].

### 3.4. Physical Characteristics of IPE

#### 3.4.1. pH Value

IPE (20 mg) was dissolved in 10 mL distilled water, methanol, DMSO, or 50% propylene glycol and the pH values of IPE in different solvents were measured using a pH meter.

#### 3.4.2. Absorption Spectrum of IPE

IPE (2 mg) was dissolved in 1 mL of methanol, DMSO, or 50% propylene glycol. The solutions were diluted in series to reach concentrations of 1,000, 500, 250, 62.5, and 31.3 μg/mL. The absorption spectrum of IPE in different solvents was measured in a microplate reader (Tecan, Austria) at wavelengths ranging from 200 to 700 nm. Methanol, DMSO, and 50% propylene glycol were used as blanks.

### 3.5. Measurement of Reducing Power

The reducing power of IPE was determined using a previously described method with slight modification [23]. Serial dilutions of IPE (50-1000 μg/mL) were prepared in 0.2 M phosphate buffer (pH 6.6) containing 1% ferrocyanate and then incubated at 50 °C for 20 minutes. Trichloroacetic acid (10%, 0.4 mL) was then added and the mixture was centrifuged at 3000 rpm for 10 minutes. The supernatant (0.5 mL) was then mixed with an equal volume of distilled water containing 10 μL of 1% ferric chloride and the absorbance was measured at 700 nm. Ascorbic acid and distilled water were used as positive and negative controls, respectively. The absorbance intensity served as a surrogate measurement of antioxidant activity of the extract as follows:

$$\mathrm{Reducing}\;\mathrm{power}\;(\%)=\left(\frac{A_{control\;at\;700\;nm}-A_{sample\;at\;700\;nm}}{A_{control\;at\;700\;nm}}\right)\times 100$$

### 3.6. DPPH Radical Scavenging Activity

In this assay, ascorbic acid was used as a positive control. Reaction mixtures containing a methanolic solution of 200 μM DPPH (100 μL) and serial dilutions of sample ranging from 50 to 1000 μg/mL (100 μL) were placed in a 96-well microplate at room temperature in the dark for 30 min. After incubation, the absorbance was read at 492 nm by an ELISA reader (Tecan, Austria). The capacity to scavenge the DPPH radical was determined by the following equation:

$$\mathrm{Scavenging}\;\mathrm{effect}\;(\%)=\left(\frac{A_{control\;at\;517\;nm}-A_{sample\;at\;517\;nm}}{A_{control\;at\;517\;nm}}\right)\times 100$$

### 3.7. Metal Chelating Activity

The chelating of ferrous ions by IPE was estimated by the ferrozine assay with slight modification [34]. Briefly, IPE (50-1000 μg/mL) was added to a solution of 2 mM FeCl_2_. The reaction was initiated by the addition of 5 mM ferrozine and the mixture was shaken vigorously and allowed to stand at room temperature for 10 min. The absorbance of the solution was then measured spectrophotometrically at 562 nm with an ELISA reader (Biotek, USA). Methanol served as the negative control and EDTA was used as the positive control. The results are expressed as a percentage of inhibition of the formation of the ferrozine-Fe^2+^ complex and were calculated by the following equation:

$$\mathrm{Chelating}\;\mathrm{effect}\;(\%)=\left(\frac{A_{control\;at\;562\;nm}-A_{sample\;at\;562\;nm}}{A_{control\;at\;562\;nm}}\right)\times 100$$

### 3.8. Determination of Hydroxyl Radical Scavenging Activity

The ability of IPE to scavenge hydroxyl radical was measured according to a previously reported method with slight modifications [35]. IPE was dissolved in methanol to reach concentrations of 50, 100, 500 and 1,000 μg/mL. Stock solutions of EDTA (1 mM), FeCl_3_ (5 mM), H_2_O_2_ (10 mM), ascorbic acid (10 mM), and deoxyribose (10 mM) were prepared in distilled water. The assay was performed by adding 100 μL of IPE, 65 μL of KH_2_PO_4_-KOH buffer (pH 7.4), 5 μL of deoxyribose, 5 μL of FeCl_3_, 5 μL of EDTA, 5 μL of H_2_O_2_, 10 μL of ascorbic acid, and 5 μL of distilled water. The mixture was then mixed and incubated at 37 °C for 1 h. After that, 100 μL of TBA (in 0.05 M NaOH containing 1 g/100 g TBA) and 100 μL of TCA (2.8 g/100 mL) were added and the mixture was incubated at 100 °C for 15 min. Then, 400 μL of butanol-pyridine solution (15:1) was added to develop the pink chromogen and the mixture was centrifuged at 4000 rpm for 10 min at 25 °C. Absorbance of the supernantant (200 µL) was then measured at 532 nm by a microplate reader (Biotek, USA). The hydroxyl radical scavenging activity of IPE is reported as percentage inhibition of deoxyribose degradation as per the following equation:

$$\mathrm{Inhibition}\;(\%)=\left(\frac{A_{control\;at\;532\;nm}-A_{sample\;at\;532\;nm}}{A_{control\;at\;532\;nm}}\right)\times 100$$

### 3.9. Peroxide Scavenging Assay

The ability of IPE to scavenge H_2_O_2_ was determined spectrophotometically as previously described [36]. A 20 mM solution of H_2_O_2_ was prepared in PBS (pH 7.4), added to various concentrations of IPE that had been dissolved in methanol (50, 100, 500 and 1,000 μg/mL), and then allowed to stand at room temperature in the dark for 10 min. The absorption was measured at 230 nm in an ELISA reader (Tecan, Austria). The H_2_O_2_ scavenging activity of IPE was determined by the following equation:

$$\mathrm{Scavenging}\;\mathrm{effect}\;(\%)=\left(\frac{A_{control\;at\;230\;nm}-A_{sample\;at\;230\;nm}}{A_{control\;at\;230\;nm}}\right)\times 100$$

### 3.10. AAPH-Induced Hemolysis Assay

Blood was obtained from male Sprague-Dawley rats *via* cardiopuncture and placed in tubes containing EDTA. This animal study adhered to *The Guidebook for the Care and Use of Laboratory Animals* (published by The Chinese Society for Laboratory Animal Science, Taiwan). The *in vitro* resistance of intact red blood cells to oxidation was evaluated with AAPH as described previously [31]. The erythrocytes were isolated by centrifugation at 3000 × *g* for 10 min, washed four times with PBS, and then re-suspended to the desired hematocrit level using the same buffer. In order to induce free radical chain oxidation in the erythrocytes, aqueous peroxyl radicals were generated by thermal decomposition of AAPH in oxygen. An erythrocyte suspension was incubated with PBS (control) and preincubated with IPE (10-500 μg/mL) at 37 °C for 30 min, followed by incubation with or without 300 mM AAPH in PBS at pH 7.4. This reaction mixture was shaken gently while being incubated for a fixed interval at 37 °C. A 200-µL aliquot of the reaction mixture was removed and centrifuged, and the absorbance of the supernatant was measured at 540 nm. Reference values were determined using the same volume of erythrocytes in a hypotonic buffer. The extent of hemolysis was calculated using the formula:

Hemolysis (%) = [(A_sample_ / A_control_)] × 100

### 3.11. Fluorescence Assay of Intracellular ROS

The fluorescence assay was performed as previously described with minor modifications [37]. Briefly, the assay is based on the use of an established nonfluorescent (DCFDA)/fluorescent (DCF) system that measures ROS. ROS are in turn responsible for the generation of fluorescence. For the fluorescence assay, human foreskin fibroblasts (Hs68) were maintained in DMEM supplemented with 10% FBS, 100 U/mL penicillin, and 100 U/mL streptomycin at 37 °C in 5% CO_2_ humidified air. The cells were subcultured following trypsinization and cells were used in the 20^th^ to 35^th^ passages. The Hs68 cells were seeded in 24-well plate at a density of 10^5^ cells/well for 24 h, rinsed once with 0.5 mL PBS and then exposed to UVB irradiation (302 nm, CL-1000M, UVP, USA). The UVB irradiation dose was 80 mJ/cm^2^ (exposure time was about 30 seconds) [17]. After that, PBS was removed, and then various concentrations of IPE (1, 5, 10 and 50 μg/mL) that had been prepared in serum-free DMEM were added and then incubated at 37 °C for 24 h. The cells were rinsed twice with 0.5 mL PBS and then incubated at 37 °C for 30 min in the presence of 10 μM DCFDA that had been prepared in DMEM. After that, the DMEM was removed and the cells were washed twice with 0.5 mL PBS. The cells were then covered with 0.5 mL PBS. Images were observed under a fluorescence microscope (Leica DMIL, German), and the fluorescence (emission 488 nm, excitation 520 nm) was measured using a microplate reader (Thermo Electron Corporation, Vantaa, Finland).

$$\mathrm{Relative}\;\mathrm{fluorescence}\;(\%)=\left(\frac{A_{control}-A_{sample}}{A_{control}}\right)\times 100$$

### 3.12. Statistical Analysis

Data are represented as the mean ± SD of at least three separate experiments. Differences between groups were analyzed by ANOVA followed by the *Scheffe’s* test. A *P* value < 0.05 was considered to indicate statistical significance.

## 4. Conclusions

Excessive production and accumulation of free radicals can accelerate the peroxidation of lipids in biosystems. Our findings indicate that IPE is a powerful absorber and neutralizer of free radicals, indicating that it is a potential anti-aging and anti-photoaging agent.
